# Cannabinoid Receptors and Glial Response Following a Basal Forebrain Cholinergic Lesion

**DOI:** 10.1021/acsptsci.2c00069

**Published:** 2022-08-04

**Authors:** Alberto Llorente-Ovejero, Iker Bengoetxea de Tena, Jonatan Martínez-Gardeazabal, Marta Moreno-Rodríguez, Laura Lombardero, Iván Manuel, Rafael Rodríguez-Puertas

**Affiliations:** † Department of Pharmacology, University of the Basque Country (UPV/EHU), Leioa 48940, Spain; ‡ Neurodegenerative Diseases, Biocruces Bizkaia Health Research Institute, Barakaldo 48903, Spain

**Keywords:** microglia, neuroinflammation, basal forebrain cholinergic lesion, rat model, Alzheimer’s disease, radioligand binding

## Abstract

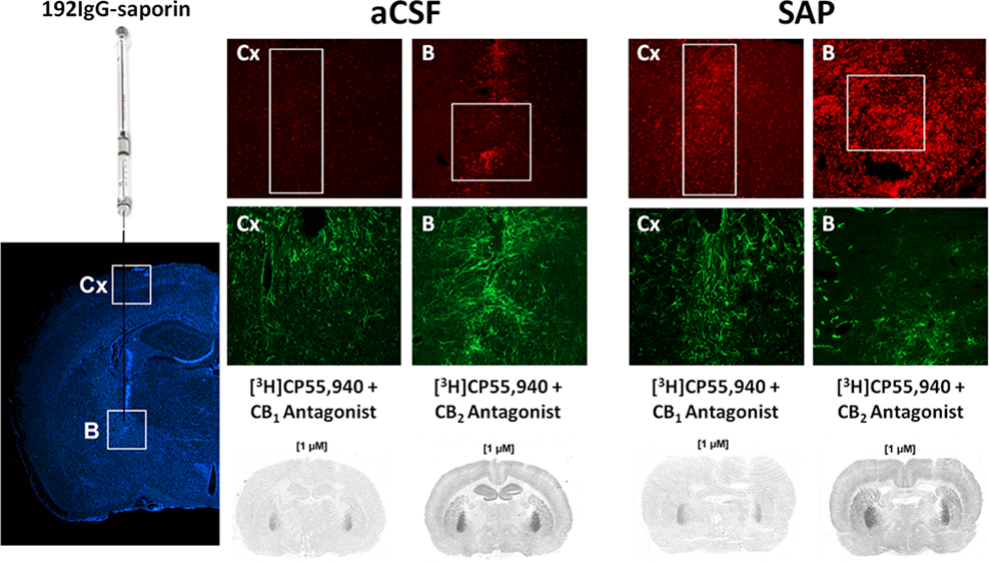

The endocannabinoid system modulates learning, memory, and neuroinflammatory processes, playing a key role in neurodegeneration, including Alzheimer’s disease (AD). Previous results in a rat lesion model of AD showed modulation of endocannabinoid receptor activity in the basalo-cortical pathway following a specific lesion of basal forebrain cholinergic neurons (BFCNs), indicating that the glial neuroinflammatory response accompanying the lesion is related to endocannabinoid signaling. In this study, 7 days after the lesion, decreased astrocyte and increased microglia immunoreactivities (GFAP and Iba-1) were observed, indicating microglia-mediated neuroinflammation. Using autoradiographic studies, the density and functional coupling to G-proteins of endocannabinoid receptor subtypes were studied in tissue sections from different brain areas where microglia density increased, using CB_1_ and CB_2_ selective agonists and antagonists. In the presence of the specific CB_1_ receptor antagonist, SR141716A, [^3^H]CP55,940 binding (receptor density) was completely blocked in a dose-dependent manner, while the selective CB_2_ receptor antagonist, SR144528, inhibited binding to 25%, at best. [^35^S]GTPγS autoradiography (receptor coupling to G_i/0_-proteins) evoked by CP55,940 (CB_1_/CB_2_ agonist) and HU308 (more selective for CB_2_) was abolished by SR141716A in all areas, while SR144528 blocked up to 51.8% of the coupling to G_i/0_-proteins evoked by CP55,940 restricted to the nucleus basalis magnocellularis. Together, these results demonstrate that there are increased microglia and decreased astrocyte immunoreactivities 1 week after a specific deletion of BFCNs, which projects to cortical areas, where the CB_1_ receptor coupling to G_i/0_-proteins is upregulated. However, at the lesion site, the area with the highest neuroinflammatory response, there is also a limited contribution of CB_2_.

Alzheimer’s disease (AD), the most common neurodegenerative disorder worldwide, progressively impairs memory and cognition skills in patients. During the progression of AD, there is a marked reduction in the density of muscarinic acetylcholine receptors in areas of the brain related to the processing and storage of memory, such as the hippocampus and the entorhinal cortex.^1^ The early impairment of basal forebrain cholinergic neurons (BFCNs) plays a key role in the development of the initial clinical symptoms of AD^2,3^ as a consequence of the specific vulnerability of these cells in the pathways that control learning and memory.^4^ Thus, animal models with a specific lesion of BFCNs are adequate for the study of dementia symptoms related with the prodromal stages of AD. The 192IgG-saporin lesion model in rat, first described in 1991,^5^ causes cognitive deficits in memory tasks such as the novel object recognition test, Morris water maze, and passive avoidance.^6,7^ Consequently, it has been used in numerous studies as a model of memory impairment and neurodegeneration.^8−10^

Besides the cholinergic system, other neuromodulatory systems are also altered during the initial stages of the disease, such as the endocannabinoid (eCB) system.^11^ The modulation of the eCB system could be a response to previous brain impairment, for example, on the BFCNs, to exert neuroprotective action.^12^ Interestingly, in the prodromal and advanced stages of AD, as studied in the 3xTg-AD mice model, the coupling to G_i/0_-proteins of the most abundant eCB receptor in the brain, CB_1_, is upregulated in areas such as the anterior thalamus but downregulated in the basal forebrain,^13^ where there is early impairment of BFCNs, suggesting a crosstalk between cholinergic and eCB systems.^14,15^ In the same line, CB_1_ receptor expression is reduced in the hippocampus of the 5xFAD mice model of AD, which show altered anxiety-like behavior and memory.^16^ Similarly, deficiency of this receptor led to enhanced cognitive impairment in the APP23 AD model, which showed reduced amyloid plaque deposition.^17^ Moreover, low doses of the CB_1_ receptor agonist, arachidonyl-2-chloroethylamide, reduced cognitive impairment in AβPP/PS1 mice when administered at a pre-symptomatic stage, pointing to a potential therapeutic use of these compounds for the treatment of AD.^18^

The regulation of neurotransmission under neurodegenerative conditions modulates eCB signaling, but the contribution of the two main cannabinoid receptors, CB_1_ and CB_2_, remains to be elucidated. There is evidence about the involvement of CB_1_ cannabinoid receptor in both patients and animal models of AD,^19,20^ but the role of the other main cannabinoid receptor, CB_2_, is not yet well understood.

The limited availability of specific and selective drugs has hindered the pharmacological study and characterization of CB_2_ receptors. Some of the most specific existing ligands include JWH-133 and AM630 (agonist and antagonist, respectively), as well as HU308 and SR144528, which were the drugs used in the present study. Similarly, questions have been raised about the specificity of antibodies targeting CB_2_.^21^ These receptors were once identified and cloned from the leukemic cell line HL-60 and were referred to as “peripheral” eCB receptors, thought to be not expressed in the brain.^22^ They were identified in circulating immune system cells, such as macrophages, and were consequently thought to intervene in some of the immune effects mediated by cannabinoids. While the involvement of CB_2_ receptors in immune response and the regulation of pain has been widely reported,^23,24^ more recent studies have also indicated the presence of this receptor in the central nervous system (CNS).^25,26^ However, doubts remain concerning its exact location and the extent of its expression throughout the CNS.^27^ While its expression in neurons is a matter of controversy, the presence of the CB_2_ receptor in microglia and its regulatory role in neuroinflammatory processes have been more widely reported.^27,28^

In neuroinflammation, immune glial cells resident in the brain, mainly microglia, react as a result of the release of several pro-inflammatory factors. These processes are common to several neurodegenerative disorders, such as AD, Parkinson’s disease, and traumatic brain injury (TBI), among others.^29^ Microglia maintains a balance between different phenotypes, some of which are pro-inflammatory, the M1 phenotype, and other anti-inflammatory, the M2 phenotype. When there is damage, microglia polarize to the M1 phenotype to perform essential functions to protect the organism and limit further harm,^30^ but a chronic activation of this phenotype can be detrimental.^31^ However, nowadays, there is evidence indicating that, under inflammatory conditions, microglia exist across a diverse spectrum of functional states that go well beyond the above-mentioned M1 and M2 phenotypes.^32,33^ CB_2_ was isolated by experiments based on PCR studies from myeloid cells,^22^ but it was also described in macrophage populations in the spleen and in leukocytes.^34^ Reported increases in the expression of this receptor subtype in AD under neuroinflammatory conditions,^35^ as well as correlation between CB_2_ expression levels and molecular markers for AD, such as Aβ_42_ levels and senile plaque score,^36^ raise the question of the therapeutic potential of CB_2_ agonists. Using a mild TBI model, presenting high axonal injury and activation of microglia, CB_2_ was upregulated and treatment with selective inverse agonist SMM-189 produced beneficial anti-inflammatory effects.^37^ In several neuropathologies that are accompanied by neuroinflammation, including multiple sclerosis (MS), Down’s syndrome, and viral encephalitis, there is selective overexpression of CB_2_ in microglia,^38^ further supporting this hypothesis. This has sparked interest in the role played by this eCB receptor subtype in the CNS, suggesting that CB_2_ could be an interesting target for treating different neurodegenerative diseases, mainly those that present neuroinflammation.

Therefore, the present study explores the coupling to G_i/0_-proteins of eCB receptors under neuroinflammatory conditions using a rat lesion model which mimics the early degeneration of BFCNs that is characteristic of AD and leads to dementia symptoms. Seven days after a specific lesion of BFCN, increased microglia density was measured at the lesion site but also at hippocampal and cortical projection areas, potentially indicating a neuroinflammatory process following the lesion that is in line with the neuroinflammation observed in the brains of AD patients. Through the combination of functional autoradiography with immunofluorescence and receptor binding assays, we explored the contribution of CB_1_ and CB_2_ receptors to the regulation of neuroinflammation in this rat model of AD.

## Results and Discussion

BFCNs constitute the main source of cholinergic innervations to the human cortex,^3^ and consequently, the progressive degeneration of these cells constitutes a key element in understanding the development of dementia symptoms that are characteristic of AD.^2^ Therefore, we have used a rat model with a specific BFCN lesion in an attempt to emulate the irreversible cholinergic impairment associated to AD. Our group previously published, for the first time, that this specific model of BFCNs using 192IgG-saporin shows modulations of eCB and cholinergic receptor coupling to G_i/0_-proteins, together with learning and memory deficits.^19^ In addition, there is a well-described microglia-mediated neuroinflammatory component in AD,^39^ but the role of both eCB receptor subtypes in the reduction of this neuroinflammation is still under discussion.^40^

In the present study, after the specific BFCN lesion, we have characterized the involvement of CB_1_ and CB_2_ in neuroinflammation in different brain areas, including the lesion area at the nucleus basalis magnocellularis (B) and the innervated hippocampal and cortical areas.

### [^3^H]CP55,940 Radioligand Binding Inhibition by CB_1_ and CB_2_ Antagonists in Different Tissues

First, using radioligand binding assays, we analyzed the affinity of CB_1_ and CB_2_ agonists and antagonists in different tissues.

**Figure 1:**
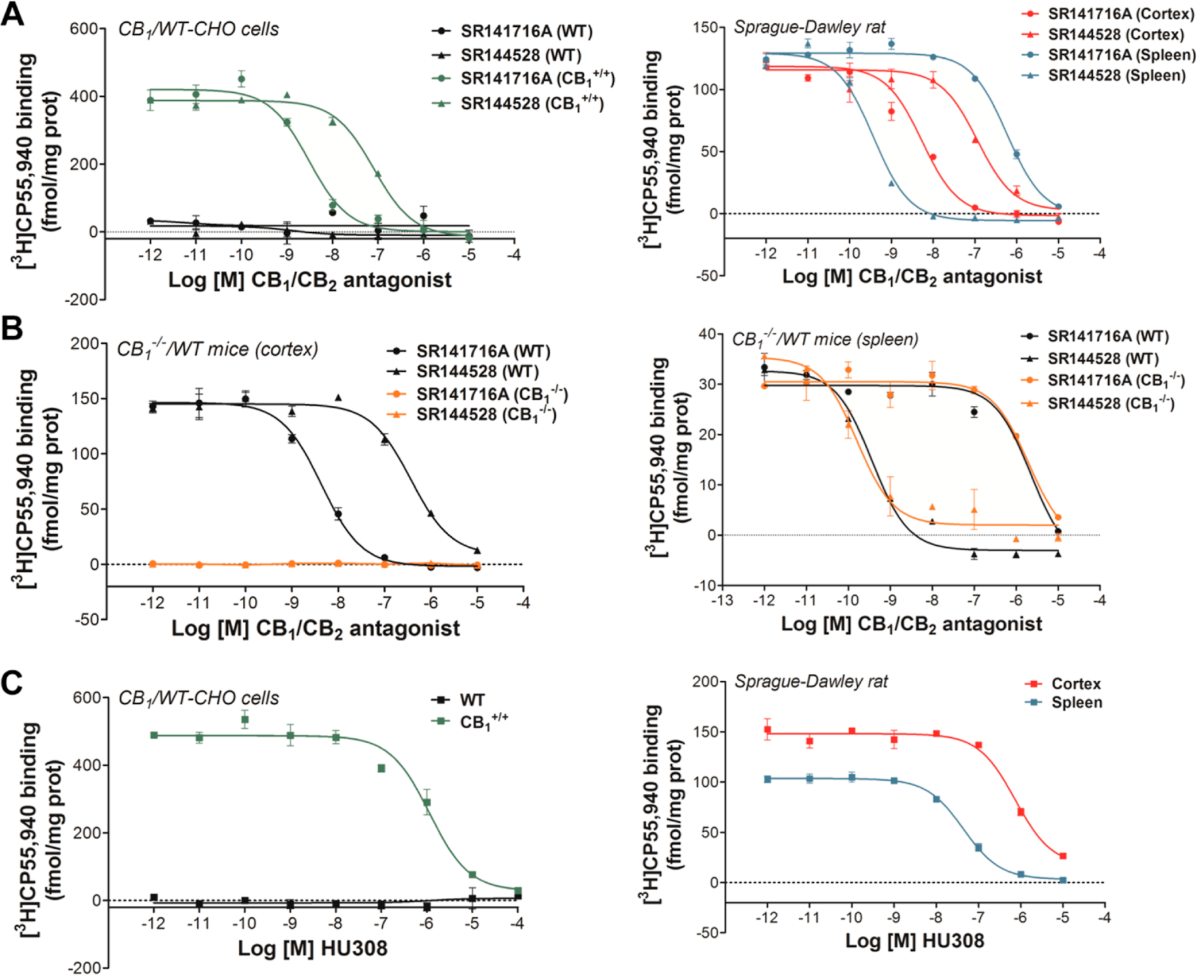
[^3^H]CP55,940 radioligand binding assays showing the displacement evoked by (A) antagonists for CB_1_ and CB_2_ receptors in CB_1_-overexpressing and matched WT control screen CHO cells and brain cortex and spleen membranes from Sprague-Dawley rats (*n* = 2), (B) antagonists for CB_1_ and CB_2_ receptors in cortical and spleen membrane homogenates from CB_1_^–/–^ mice (*n* = 2) and matched WT (*n* = 2), and (C) specific agonist for CB_2_ receptors HU308 in CB_1_-overexpressing and matched WT control CHO cells and cortical and spleen membrane homogenates from Sprague-Dawley rats (*n* = 2).

Both CB_1_ and CB_2_ antagonists, SR141716A (rimonabant) and SR144528, were able to inhibit the binding of [^3^H]CP55,940 in CB_1_-overexpressing membranes, with *K*_i_ values of 10^–8.8^ and 10^–7.4^ M, respectively (see Figure 1A). Similarly, both antagonists also displaced [^3^H]CP55,940 binding in cortical membranes from Sprague-Dawley rats, with *K*_i_ values of 10^–8.6^ and 10^–7.2^ M (see Figure 1A). As expected, SR141716A showed a higher affinity in both CB_1_-overexpressing cells and rat cortical membranes. In purified membranes taken from rat spleens, which have a high density of CB_2_ receptors and absence of CB_1_ receptors,^16^*K*_i_ values were 10^–6.5^ and 10^–9.7^ M (see Figure 1A), respectively. In this tissue, SR144528 behaved like a high-affinity competitive compound against [^3^H]CP55,940 binding.

In cortical membranes from the CB_1_^–/–^ mice, both antagonists failed to displace [^3^H]CP55,940 binding, as opposed to results obtained in matched wild-type (WT) controls, in which *K*_i_ values were 10^–8.6^ and 10^–6.7^ M (see Figure 1B). In spleen membranes from the CB_1_^–/–^ mice, *K*_i_ values were 10^–6.0^ and 10^–10.1^ M, indicating the different affinities of both antagonists for CB_2_ receptors (see Figure 1B). Together, these results coincide with previous studies in indicating that CP55,940 is a nonselective CB_1_/CB_2_ agonist^41^ and that SR141716A is a selective antagonist for CB_1_.^42^ Regarding SR144528, its selectivity for CB_2_ receptors has been reported before,^43^ but our results indicate that, at concentrations over 10^–7^ M, it binds to CB_1_ receptors as well.

In binding assays performed in the presence of a more selective agonist for CB_2_ receptors, HU308, *K*_i_ values were 10^–6.2^ M in CB_1_-overexpressing cells and 10^–6.4^ M in rat cortical membranes and 10^–7.6^ M in rat spleen membranes (see Figure 1C), respectively. The fact that HU308 inhibited [^3^H]CP55,940 with a higher affinity in spleen membranes compared to brain cortex and CB_1_-overexpressing membranes is indicative of its higher affinity for CB_2_ receptors. HU308 has previously been described as a selective agonist for CB_2_ receptors, showing a 278-fold higher affinity for CB_2_ than for CB_1_.^44^ We indeed provide evidence that HU308 is an appropriate pharmacological tool for the study of CB_2_ in tissues with a high density of these receptors, such as the spleen, but that it is not specific at concentrations over 10^–6^ M in tissues with a high density of CB_1_ receptors, such as the cortex.

### Iba-1 and GFAP Immunoreactivity and [^3^H]CP55,940 Binding in the Presence of CB_1_ and CB_2_ Antagonists in a Rat Lesion Model of AD

With the pharmacological tools previously characterized (see Figure 1), we studied the role of both CB_1_ and CB_2_ receptors in the neuroinflammatory process accompanying a BFCN lesion.

**Figure 2:**
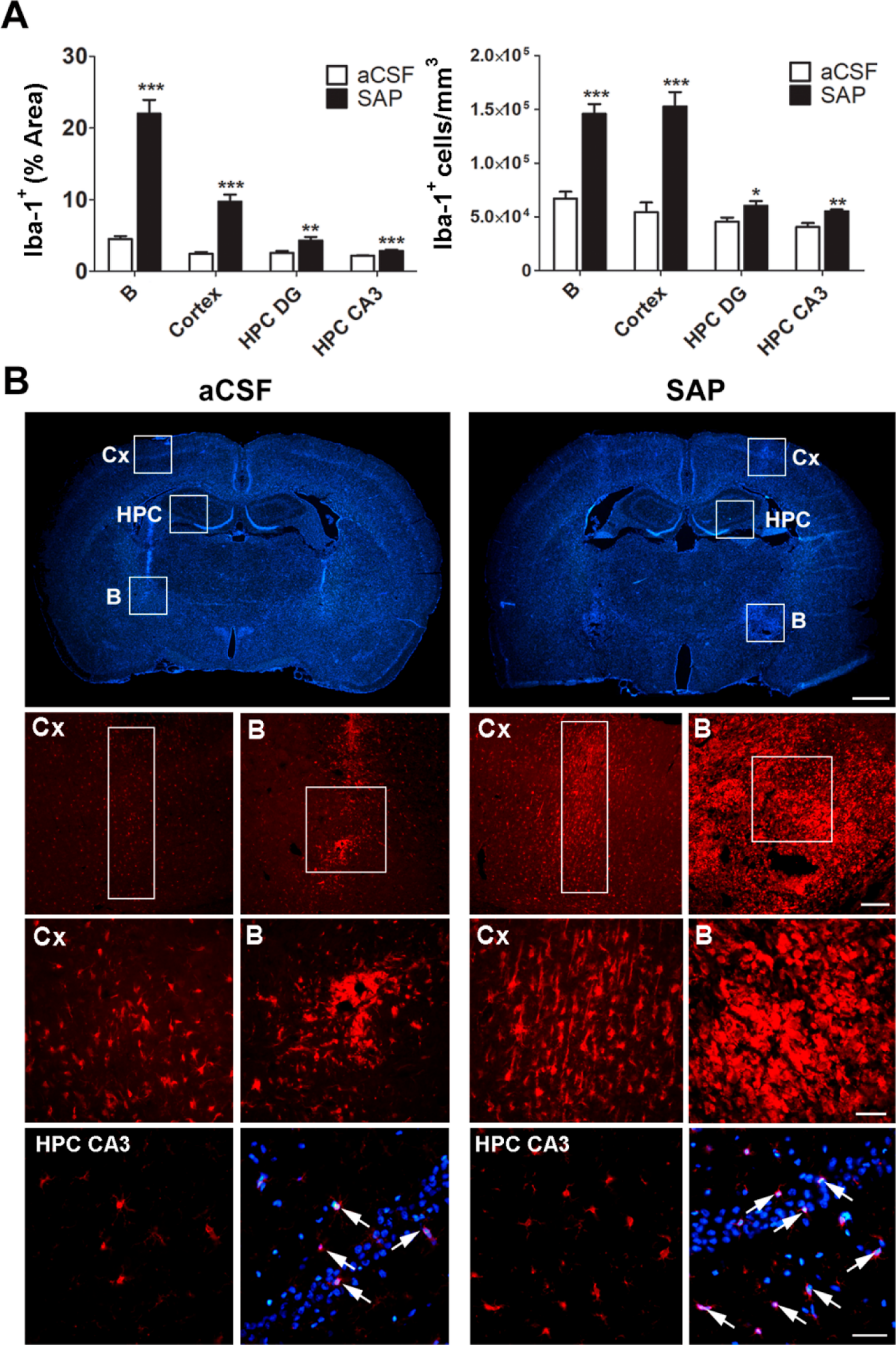
(A) Microglial cell immunoreactivity in different brain areas measured as the percentage of fluorescent brain area labeled with Iba-1 antibody (left) and the number of microglial cells per mm^3^ of tissue (right). *p < 0.05, **p < 0.01, and ***p < 0.001, aCSF (*n* = 6) vs SAP (*n* = 6). (B) Top photographs correspond to 25-fold magnification images (scale bar = 2 mm) of Hoechst-stained slices of aCSF and SAP Sprague-Dawley rats. Squares indicate the areas that were analyzed: Cx, HPC DG, HPC CA3, and B. Photographs in the middle correspond to 100-fold (scale bar = 200 μm) and 400-fold (scale bar = 50 μm) magnification images of the immunosignal associated to microglial cells (Iba-1) in the areas analyzed: Cx, HPC CA3, and B. Squares and rectangles indicate the ROIs in which the photographs used for the analysis were randomly taken. Photographs at the bottom correspond to 400-fold magnification (scale bar = 50 μm) merged images of Hoechst and Iba-1 staining, where individual microglial cells are depicted (arrows). Cortex: Cx; nucleus basalis magnocellularis: B; hippocampus dentate gyrus: HPC DG; Hippocampus CA3 area: HPC CA3.

The presence of microglia, measured as the percentage of brain area stained with the Iba-1 antibody and as the number of microglial cells per mm^3^ of tissue, significantly increased in the group of animals with the BFCN lesion using 192IgG-saporin (SAP group) in the lesioned area, B, as well as in other innervated areas far from the focus: the cortex and the hippocampus (see Figure 2). Given the difficulty and current lack of consensus regarding protein markers for the detection of the different states of microglia,^45^ we have preferred to avoid such descriptions in the present work, focusing instead on describing the increased Iba-1 immunoreactivity in the different brain areas.

**Figure 3:**
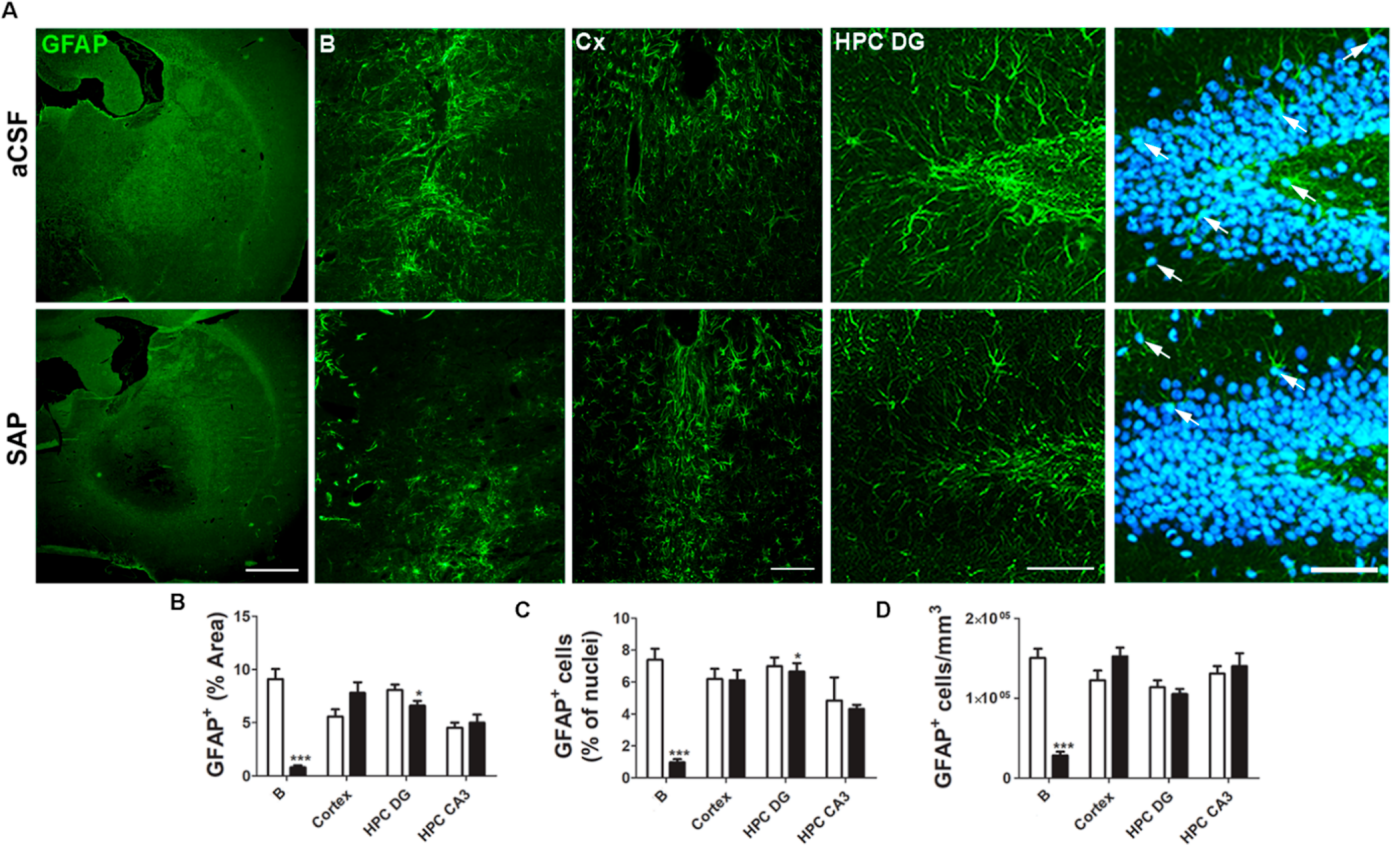
(A) Photographs on the first column corresponding to 25-fold magnification images of aCSF and SAP Sprague-Dawley rats immunolabeled for GFAP (scale bar = 2 mm). Photographs on the second and third columns correspond to 200-fold magnification GFAP immunopositive images from B and Cx, respectively (scale bar = 200 μm). In the fourth column, representative images from HPC DG immunolabeled for GFAP showing the abundance of astrocytes in the vehicle and lesioned animals (scale bar = 50 μm) are shown. In the fifth column are representative images from HPC DG merging of Hoechst and GFAP staining (scale bar = 50 μm), where individual astrocytes are depicted (arrows). The histograms show the (B) GFAP-immunopositive area, the (C) percentage of astrocytes out of the total amount of cell nuclei, and the (D) number of astrocytes per mm^3^ of tissue in aCSF (white, *n* = 6) and SAP (black, *n* = 6) groups of rats. *p < 0.05 and ***p < 0.001, aCSF vs SAP. Cortex: Cx; nucleus basalis magnocellularis: B; hippocampus dentate gyrus: HPC DG; Hippocampus CA3 area: HPC CA3.

In contrast, the presence of astrocytes, measured as the percentage of brain area stained with the GFAP antibody, the percentage of astrocytes out of the total amount of cell nuclei, and the number of astrocytes per mm^3^ of tissue, was significantly reduced in the SAP group in the lesion area, B, and, to a lesser extent, in the dentate gyrus (see Figure 3). Together, these results clearly indicate a neuroinflammatory process following the lesion, specifically driven by microglial cells. However, previous studies performed using 192IgG-saporin reported a breach in the blood–brain barrier following the lesion,^46^ suggesting that macrophage recruitment to the lesion site from peripheral tissues is a possibility. While microglia are divergent from peripherally infiltrated macrophages, their precise discrimination is surprisingly difficult as a consequence of shared cellular markers, such as the one used in this study, Iba-1.^45^ The discrimination between these two cellular types should be analyzed in further studies with that specific aim. In this work, for a better understanding of our results, we will refer to Iba-1+ cells as microglia, which are likely to represent most of this immunostaining.

The presence of microglia in a context of neuroinflammation was expected but decreased astrocyte immunoreactivity was not. Previous studies have demonstrated that astrocytes migrate and/or proliferate after an acute brain lesion,^47^ such as a stroke.^48^ In our lesion model, the observed decrease in astrocyte immunoreactivity may be a consequence of the lesion itself, since the 192IgG-saporin toxin used is specifically directed to cells expressing the p75^NTR^ receptor, which include cholinergic neurons, but also astrocytes in response to certain insults, such as seizures.^49^ In fact, upregulation of the p75^NTR^ receptor in the lesion site following a cortical stab wound promotes astrocyte proliferation as a response to damage.^50^ That process might be happening in our model following the neuronal death caused by 192IgG-saporin. Astrocytes in the lesioned area might express p75^NTR^ as a response to the lesion, becoming vulnerable themselves to the effects of the toxin. Further studies are needed to explain the mechanism for inducing this paradoxical decrease in astrocyte immunoreactivity following the lesion.

**Figure 4:**
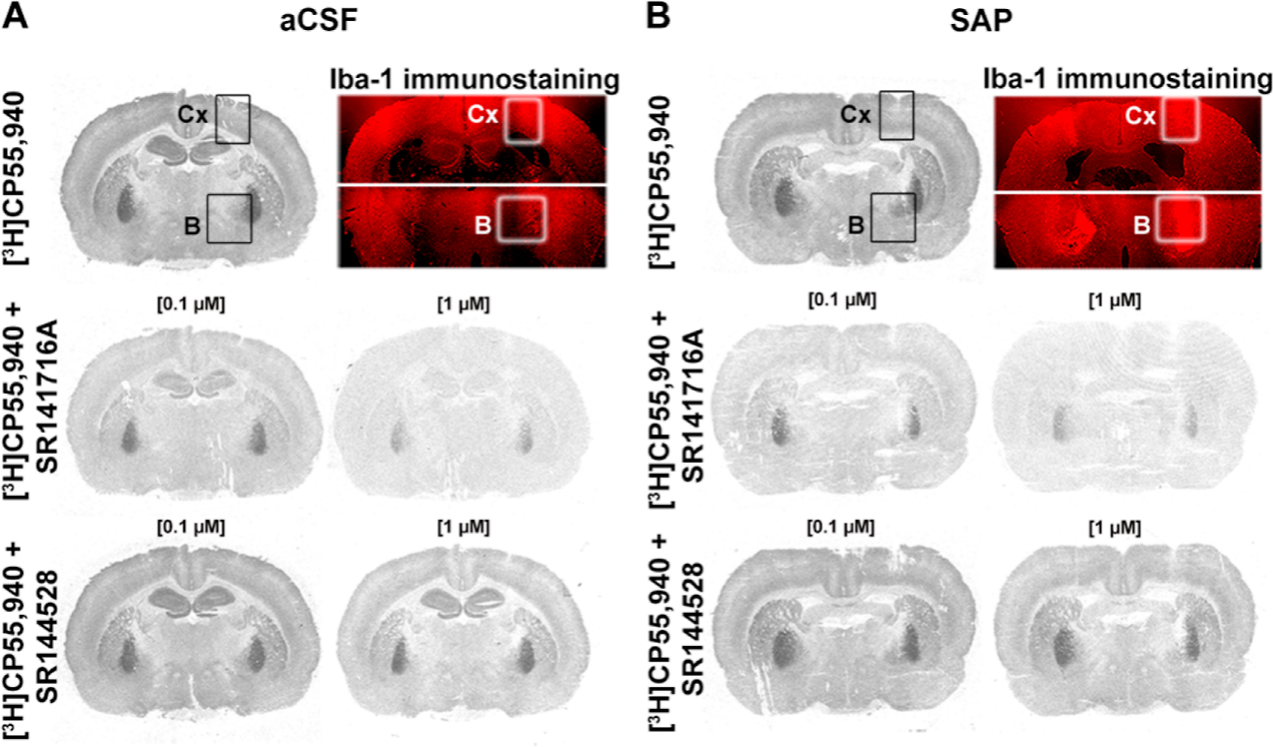
Autoradiograms (gray scale) showing the total distribution of cannabinoid receptors ([^3^H]CP55,940 binding) and in the presence of specific antagonists SR141716A and SR144528 in consecutive brain sections from (A) aCSF and (B) SAP Sprague-Dawley rats. The immunosignal (red) associated with microglial cells (Iba-1) is shown. The areas used for measurements on Cx and B are indicated (squares). Cortex: Cx and nucleus basalis magnocellularis: B.

In the areas where microglia immunoreactivity increased, mainly B and the cortex, receptor autoradiography experiments showed a marked reduction of [^3^H]CP55,940 binding in the presence of the specific CB_1_ antagonist SR141716A. This reduction in [^3^H]CP55,940 binding was not observed in the presence of the specific CB_2_ antagonist SR144528 (see Figure 4). SR141716A was able to inhibit around 60% of [^3^H]CP55,940 binding with a concentration of 0.1 μM and 100% of the binding with 1 μM, while SR144528 was able to displace just 25% of the binding, at best, with a concentration of 1 μM (see Figure S1). In the context of microglia-mediated neuroinflammation, we expected an overexpression of CB_2_ receptors, as it has been reported following similar brain insults. For example, in a model of hypoxic-ischemic brain damage following middle cerebral artery occlusion, CB_2_ was expressed in brain-resident microglia 3 days after surgery,^51^ and in an in vivo model of Parkinson’s disease, increased CB_2_ receptor expression was accompanied by an upregulation of MAC-1, a marker of microglia, in the same brain region.^52^ In AD samples from postmortem human brains, CB_2_ receptor immunoreactivity was found exclusively in grouped cells around neuritic-plaques, which showed morphological properties characteristic of microglia.^35^ Similarly, CB_2_ expression was augmented in frontal cortex samples from AD patients.^36^

The present pharmacological study was performed in a rat lesion model of AD, which develops microgliosis not only at the lesion site but also at cortical and subcortical projection areas 7 days after the lesion, comparable to the widespread neuroinflammation caused by AD pathology. In previous autoradiographic studies analyzing the specific binding of [^3^H]CP55,940, our group described an increase in cannabinoid receptor density in B following the specific BFCN lesion,^19^ coincident with the present study (see Figure S2). These results are in line with previous studies, which have shown overactivation of the eCB system during the early stages of AD.^11^ Here, we aimed to elucidate the contribution of CB_1_ and CB_2_ receptors to this increased cannabinoid receptor density. Under the same experimental conditions previously used,^13^ SR141716A (1 μM) abolished [^3^H]CP55,940 binding in all the analyzed brain areas, while SR144528 only reached 25% of inhibition. These results suggest that increased [^3^H]CP55,940 binding, including in the brain areas showing microgliosis, corresponds mainly to the CB_1_ receptor subtype.

### CB_1_ and CB_2_ Coupling to G_i/0_-Proteins in the Presence of Specific Antagonists in Areas with Increased Microglia Immunoreactivity in a Rat Lesion Model of AD

**Figure 5:**
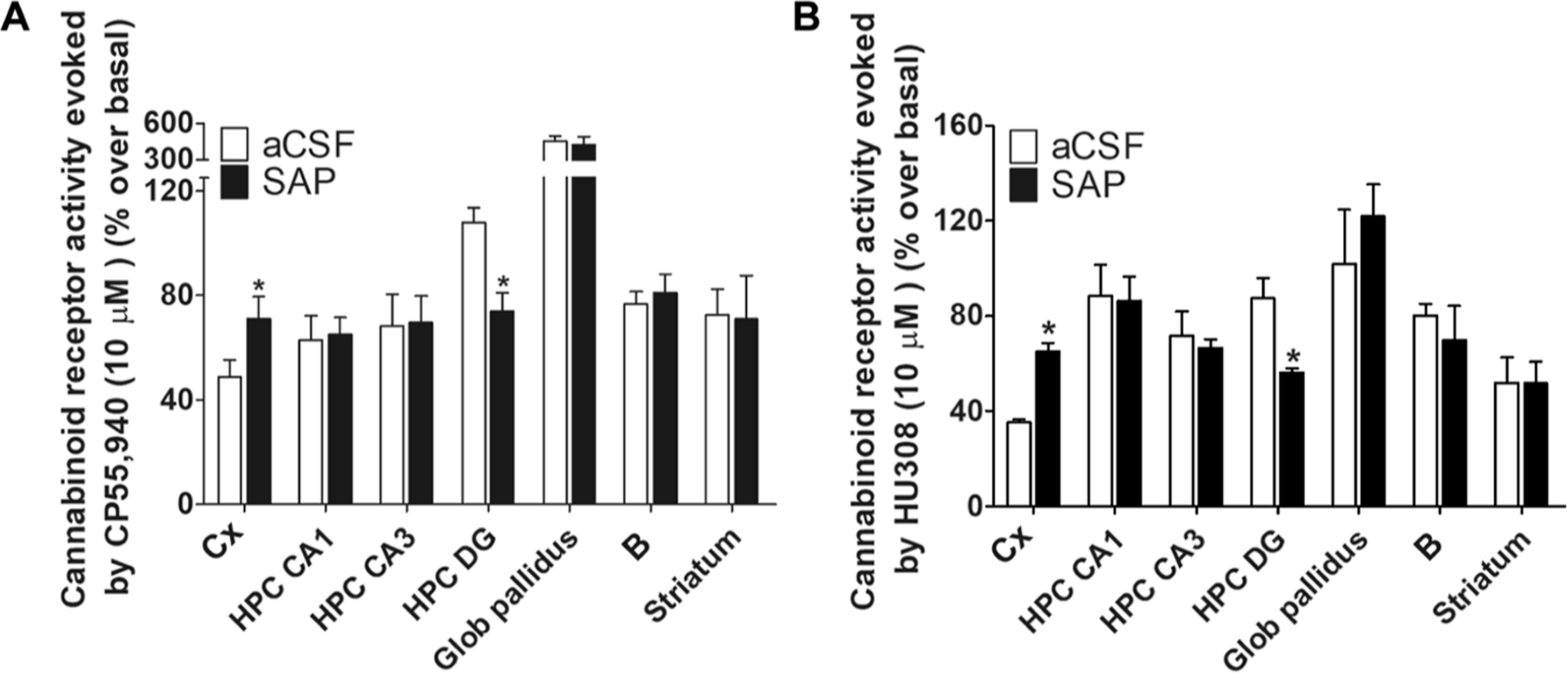
[^35^S]GTPγS binding evoked by (A) CB_1_/CB_2_ agonist CP55,940 and (B) CB_2_ agonist HU308 shown as the percentage of coupling to G_i/0_-proteins over basal in each brain area, in aCSF (*n* = 5) and SAP (*n* = 5) Sprague-Dawley rats. *p < 0.05, aCSF vs SAP. Cortex: Cx; nucleus basalis magnocellularis: B; hippocampus dentate gyrus: HPC DG; hippocampus CA3 area: HPC CA3; hippocampus CA1 area: HPC CA1; Glob pallidus; Globus pallidus.

To further characterize the functional state of G_i/0_-coupled cannabinoid receptor-mediated signaling in our model of AD, we analyzed CB_1_ and CB_2_ coupling to G_i/0_-proteins in different brain areas. For these experiments, we used CB_1_/CB_2_ agonist CP55,940 and more selective CB_2_ agonist HU308.^44^ We decided to use the same agonist used to measure CB_1_/CB_2_ density, CP55,940, instead of WIN55,212-2, used in our previous study,^19^ where we observed a slight increase in [^35^S]GTPγS binding (receptor coupling to G_i/0_-proteins) in the SAP group in B. In the present study, we did not observe such a statistically significant increase (see Figure 5). To explain these divergent results, we performed a study specifically aimed at comparing the coupling to G_i/0_-proteins of both cannabinoid receptor agonists, WIN55,212-2 and CP55,940, using [^35^S]GTPγS binding autoradiography. We do not report significant differences between the stimulation evoked by these two different agonists, but in every area analyzed (including B), there is a tendency indicating that WIN55,212-2 stimulates more than CP55,940 when used at the same concentration (see Figure S3), which could account for the minor discrepancy observed between both studies.

Similar stimulations were measured in the presence of CP55,940 and HU308 in most of the areas analyzed, including the cortex, hippocampus, and B. Importantly, stimulation evoked by CP55,940 was significantly higher in the *globus pallidus* (see Figure 5), which has one of the highest densities of CB_1_ receptors in the brain.^53^

Together, these results suggest that, in areas where we have determined that microglia immunoreactivity increased following the lesion, the expected increase in the coupling to G_i/0_-proteins evoked by HU308 related to microglial CB_2_ was not observed. At the minimum concentration required for these experiments (10 μM),^20,54−56^ the coupling to G_i/0_-proteins evoked by HU308 seems to correspond mainly to the CB_1_ receptor subtype. This is in line with a previous study also using SR141716A and HU308, which reports that WIN55,212-2, a CB_1_/CB_2_ receptor agonist, ameliorated disease progression in a mouse model of MS, exerting CB_1_-mediated anti-inflammatory effects^57^ and another study indicating that, in the 5xFAD mice model of AD, CB_1_ blockade exacerbated inflammation.^58^

**Figure 6:**
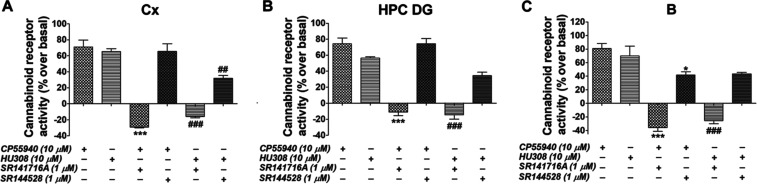
[^35^S]GTPγS binding evoked by CB_1_/CB_2_ agonist CP55,940 and CB_2_ agonist HU308 shown as the percentage of coupling to G_i/0_-proteins over basal, in SAP (*n* = 5) Sprague-Dawley rats, in the presence of specific antagonists for CB_1_ and CB_2_ receptor subtypes, SR141716A and SR144528, respectively, in the (A) Cx, (B) HPC DG, and (C) B. *p < 0.05 and ***p < 0.001 vs CP55,940; ##*p* < 0.01 and ###*p* < 0.001 vs HU308. Cortex: Cx; nucleus basalis magnocellularis: B; hippocampus dentate gyrus: HPC DG.

To further determine the contribution of both eCB receptor activities in lesioned rats 7 days after surgery, functional coupling evoked by CP55,940 and HU308 was also analyzed in the presence of specific antagonists SR141716A and SR144528. In line with the results obtained in [^3^H]CP55,940 binding assays, SR141716A was able to completely block the functional coupling of cannabinoid receptors in the presence of both agonists in the three areas analyzed: the cortex, hippocampus dentate gyrus, and B (see Figure 6). Meanwhile, SR144528 was able to block only 51.8% of the [^35^S]GTPγS binding evoked by CP55,940 in B (see Figure 6C). SR144528 was able to inhibit around 50% of the stimulation evoked with a more specific CB_2_ agonist, HU308 (see Figure 6A). Together, these results indicate an absence of detectable levels of CB_2_ coupling to G_i/0_-proteins in projection areas where microglia immunoreactivity increased, but in B, where the increase in density is higher, slight CB_2_ coupling to G_i/0_-proteins was detected. These findings are in coincidence with previous data from genetic models of familial AD, such as 5xFAD mice, which show CB_2_ upregulation in areas affected by amyloid-triggered neuroinflammation.^38^ Regarding the cortex and dentate gyrus, where we did not find increased CB_2_ coupling to G_i/0_-proteins in spite of increased microglial immunoreactivity, a plausible explanation for that could be differences in the microglial activation states observed in these areas^59^ (see Figure S4).

## Conclusions

Overall, our results indicate that 1 week after a specific lesion of BFCN, which damages the same cholinergic pathways that are degenerated in the early stages of AD, the neuroinflammatory process is characterized by increased microglia and decreased astrocyte immunoreactivities. In cortical BFCN projection areas, CB_1_ receptor coupling to G_i/0_-proteins is upregulated, while at the lesion site, the area showing the highest increase of microglia, slight CB_2_ coupling to G_i/0_-proteins was detected. Several studies have suggested that both cannabinoid receptors play a key role in the regulation of neuroinflammation. However, the extent to which each one of the receptors contributes needs to be further clarified and may depend on several factors, including the type of insult, the animal model used for the study, and the temporal pattern of the inflammatory response.

## Materials and Methods

### Animals and Cell Lines

Forty-one adult male Sprague-Dawley rats (200–250 g) were used for the autoradiographic and immunochemical studies. Two additional adult male Sprague-Dawley rats, two WT C57BL/6 mice, and two CB_1_^–/–^ mice, provided by C. Ledent from the University of Brussels, were used for the binding studies. Membranes from WT and CB_1_^+/+^-overexpressing CHO cells were also used for radioligand binding assays, and tissue obtained from WT and CB_1_^–/–^ mice was used exclusively to further characterize the specificity of the ligands in radioligand binding assays. All the animals were bred and kept in makrolon cages (38.2 × 22.0 cm) under standard laboratory conditions (food and water *ad libitum*, 22 ± 2 °C, 12 h light/dark cycle, 65–70% relative humidity). The experimental protocols regarding the use of laboratory animals were approved by the University of the Basque Country Local Ethics Committee for animal research (CEEA M20-2018-52 and 54), in accordance with EU directive 2010/63/EU for animal experiments.

### Chemicals

192IgG-saporin was acquired from Millipore (Temecula, CA, USA). [^35^S]GTPγS (1250 Ci/mmol) and [^3^H]CP55,940 (131.8 Ci/mmol) were acquired from PerkinElmer (Boston MA, USA). The [^14^C]-microscales, used as standards in the autoradiographic experiments, were acquired from American Radiolabeled Chemicals (St. Louis, MO, USA). Kodak Biomax MR β-radiation-sensitive films, bovine serum albumin (BSA), dl-dithiothreitol, adenosine deaminase, guanosine 5′-diphosphate, guanosine 5′-O-3-thiotriphosphate (GTPγS), ketamine and xylazine, as well as CP55,940 were acquired from Sigma-Aldrich (St. Louis, MO, USA). SR141716A and HU308 were acquired from Tocris Bioscience (Bristol, UK) and SR144528 from Cayman-Chemicals (MI, USA). All the compounds used were of the highest commercially available quality.

### Basal Forebrain Cholinergic Lesion and Tissue Preparation

All surgery procedures were carried out under aseptic conditions. 192IgG-saporin toxin was used to selectively eliminate cholinergic neurons in the B, following the procedure previously described and verified by our group.^19^ Rats were randomly assigned either to the control group, which received artificial cerebrospinal fluid (aCSF; *n* = 20), or to a 192IgG-saporin-infused group (SAP, *n* = 21). The aCSF vehicle was prepared as follows: 0.15 M NaCl, 2.7 mM KCl, 0.85 mM MgCl_2_, and 1.2 mM CaCl_2_ (pH 7.4) were mixed and sterilized by filtration with 0.4 μm-Ø filters (EMD Millipore, CA, USA). Rats were anesthetized with ketamine/xylazine (90/10 mg/kg, i.p.) and received a bilateral intraparenchymal infusion of either aCSF or 192IgG-saporin into the B according to the following stereotaxic coordinates: −1.5 mm anteroposterior from the Bregma, ±3 mm mediolateral from the midline, and +8 mm dorsoventral from the cranial surface (Paxinos and Watson, 2005). Rats received 135 ng/μL of 192IgG-saporin (1 μL/hemisphere; 0.25 μL/min). For the control group, aCSF was injected with the same volume/rate.

On day 7 after surgery, all the animals were anesthetized with ketamine/xylazine (90/10 mg/kg; i.p.). Animals were sacrificed by decapitation to obtain “fresh tissue” (i.e., brain and spleen samples were quickly dissected and immediately placed in a −80 °C dry air atmosphere for 15 min and then covered with a parafilm and kept at −80 °C until being processed). Later, they were cut into 20 μm sections using a Microm HM550 cryostat (Thermo Fisher Scientific, Waltham, MA, USA) equipped with a freezing-sliding microtome at −25 °C and mounted onto gelatin-coated slides and stored at −25 °C until use for radioligand binding studies. The animals used for immunofluorescence studies were transcardially perfused as previously described.^19^ The extension of the BFCN lesion was verified by acetylcholinesterase staining (see Figure S5).

### Binding Assays

Brain and spleen membranes from rat, WT mice and CB_1_^–/–^ mice, as well as WT and CB_1_^+/+^-overexpressing CHO cells were used to determine the affinity of the specific antagonists for CB_1_ or CB_2_ (SR141716A or SR144528, respectively) and HU308 as a specific agonist of CB_2_ receptors. For the preparation of the membranes, the cortexes from Sprague-Dawley rats (*n* = 2), WT mice (*n* = 2), and CB_1_^–/–^ mice (*n* = 2) were dissected. The spleens from Sprague-Dawley rats (*n* = 2) were also dissected as a tissue with a high concentration of CB_2_ receptors and low in CB_1_. Tissues were homogenized following the protocol previously described.^60^ To perform the binding assays, a protein concentration of 0.05 mg/mL of WT and CB_1_^+/+^-overexpressing CHO cells was used, and a protein concentration of 0.1 mg/mL for the cortex and spleen homogenates was used. Membrane aliquots and CHO cells were resuspended in a reaction buffer (Tris-HCl 50 mM, MgCl_2_ 3 mM, EDTA 1 mM, and 1% of BSA, pH 7.4). HU308, SR141716A, and SR144528 were used in concentrations in triplicate (a minimum of two replicates from each concentration were used ×2 animals or cell homogenates), ranging from 10^–12^ to 10^–4^ M and incubated for 2 h at 37 °C with agitation in the presence of 0.5 nM of [^3^H]CP55,940. Nonspecific binding was defined as the binding of [^3^H]CP55,940 in the presence of 10^–4^ M of WIN55,212-2 using a different agonist with a similar profile of CB_1_/CB_2_ affinities for a better estimation of specific binding sites. After incubation, the reaction was stopped by adding ice-cold wash buffer (Tris-HCl 50 mM and 0.5% of BSA, pH 7.4). Then, the membranes were retained by vacuum filtration to a Whatman GF/C glass microfiber filter (Sigma-Aldrich, St. Louis, MO, USA). Filters with the bound radioligand were transferred to vials containing 5 mL of Ultima Gold cocktail (PerkinElmer, Boston, MA, USA) and measured with a Packard Tri-Carb 2200CA liquid scintillation counter (PerkinElmer, Boston, MA, USA). To determine agonist-evoked binding, nonspecific binding was subtracted from the total.

### Autoradiographic Assays

#### [^3^H]CP55,940 Receptor Autoradiography

The density of CB_1_ and CB_2_ receptors was measured in fresh frozen 20 μm sections of rats from both groups, aCSF (*n* = 8) and SAP (*n* = 8), as previously described.^14^ Briefly, tissue sections mounted on slices were first immersed in copling jars for preincubation and later incubated in the presence of the [^3^H]CP55,940 radioligand. Specific antagonists for CB_1_ and CB_2_, SR141716A (0.1 and 1 μM) and SR144528 (0.1 and 1 μM), respectively, were used to check for the specificity of the binding of each eCB receptor subtype in the consecutive slices along with the [^3^H]CP55,940 radioligand. After incubation, tissue sections were washed with preincubation buffer at 4 °C and then dipped in distilled water and dried. Sections were exposed for 21 days at 4 °C to β-radiation-sensitive films in hermetically closed cassettes. For the calibration of the optical densities to fmol/mg tissue equivalent, [^3^H]-microscales were used. The calibrated films were scanned and quantified using Fiji software (Fiji, Bethesda, MA, USA).

#### Functional [^35^S]GTPγS Autoradiography

[^35^S]GTPγS binding assays upon activation of cannabinoid receptors were assayed in fresh frozen 20 μm sections of rats from both groups, aCSF (*n* = 5) and SAP (*n* = 5), as previously described.^55^ Briefly, tissue sections mounted on slices were first immersed in copling jars for preincubation and later incubated in the presence of [^35^S]GTPγS. CB_1_/CB_2_ receptor-mediated coupling to G_i/0_-proteins was determined with CP55,940 (10 μM) or HU308 (10 μM) agonists, which was determined in previous studies as the optimal concentration for these type of experiments.^20,54−56^ Although many neurotransmitter receptors bind agonists with high affinity (*K*_d_) in the nanomolar range, micromolar concentrations of the same agonists are required to elicit a functional effect^61^ and such is the case of the [^35^S]GTPγS binding assays. Basal coupling to G_i/0_-proteins for each brain area was determined in the absence of agonists. SR141716A (0.1 and 1 μM) and SR144528 (0.1 and 1 μM) antagonists were used to check for the specificity of the [^35^S]GTPγS binding by blocking CB_1_ or CB_2_ subtypes, respectively. Nonspecific [^35^S]GTPγS binding was determined in the presence of GTPγS (10 μM). After incubation, tissue sections were washed twice in 50 mM HEPES (pH 7.4) buffer at 4 °C and dried. Sections were exposed to β-radiation-sensitive films in hermetically closed cassettes for 48 h at 4 °C. For the calibration of the optical densities to the nCi/g tissue equivalent, [^14^C]-microscales were used. The calibrated films were scanned and quantified using Fiji software. The coupling to G_i/0_-proteins ([^35^S]GTPγS binding) evoked by the agonists was expressed as the percentage over basal according to the following formula: ([^35^S]GTPγS agonist-stimulated binding) × 100/([^35^S]GTPγS basal binding)-100.

### Immunofluorescence

For the detection of microglia, 10 μm brain slices from aCSF (*n* = 6) and SAP (*n* = 6) were incubated with primary rabbit polyclonal anti-Iba-1 [1:500] antibody (Fujifilm Wako Chemicals, VA, USA). The antibody was diluted in phosphate-buffered solution (PBS) (0.1 M, pH 7.4), which contained 0.5% of BSA, and the samples were incubated overnight at 4 °C. On the following day, the samples were washed for 30 min in PBS and incubated for an additional 30 min at 37 °C with the appropriate secondary antibody. To reveal Iba-1, Cy3-labeled goat anti-rabbit [1:250] antibody (Jackson Immunoresearch, West Grove, PA, USA) was used.

For the detection of M1 microglia phenotype, 10 μm brain slices from aCSF (*n* = 3) and SAP (*n* = 3) were incubated with primary rabbit polyclonal anti-iNOS [1:250] antibody (BD Biosciences, Franklin Lakes, NJ, USA), and the same protocol was followed. To reveal iNOS, the secondary antibody was Alexa 488-labeled donkey anti-rabbit [1:200] (Thermo Fisher Scientific, Waltham, MA, USA). The M1 microglia phenotype was determined based on the colocalization of Iba-1 and iNOS immunofluorescence.

For the detection of astrocytes, brain slices from aCSF (*n* = 6) and SAP (*n* = 6) were incubated with primary mouse anti-GFAP [1:1000] antibody (Millipore, Temecula, CA, USA), and the same protocol was followed. In this case, the secondary antibody was FITC-labeled goat anti-mouse [1:80] (Jackson Immunoresearch, West Grove, PA, USA). After incubation with the secondary antibodies, all sections were incubated with Hoechst 33258 for 15 min to also label the cell nuclei, then washed for 30 min in PBS, and finally mounted with *p*-phenylendiamine–glycerol (0.1%) for immunofluorescence. For colocalization, ZEN2014 software (Carl Zeiss) was used.

### Quantification of Immunofluorescence

Immunofluorescence images were used to make an estimation of the astrocyte and microglial cell immunoreactivities following stereotaxic coordinates. 200-fold magnification (0.50 numerical aperture) photomicrographs (SPOT Flex Shifting Pixel CCD imaging camera) were acquired using an Axioskop 2 Plus epifluorescence microscope (Carl Zeiss Meditec AG, Jena, Oberkochen, Germany) in both hemispheres at three different coronal levels following Paxinos Atlas stereotaxic coordinates (Paxinos and Watson, 2005). One level included both the core portion of the B and the injection site in the cortex (Interaural 7.80 mm, Bregma −1.20 mm), another level included a more caudal portion of both the B and the cortex as well as the dorsal hippocampus (Interaural 7.08 mm, Bregma −1.92 mm), and the third one included a more caudal part of the hippocampus including the ventral portion (Interaural 4.20 mm, Bregma −4.80 mm). Regions of interest (ROIs) were selected for each brain area (Cx, B, HPC DG, and HPC CA3) and four images (two in each hemisphere) were randomly acquired at 200-fold magnification within them for quantification. Using Fiji software (NIH, Bethesda, MD, USA), images were converted into an 8-bit binary mode, and different cells were identified by applying the watershed option. The number of astrocytes (GFAP^+^-ir), microglial cells (Iba-1-ir), and nuclei (N) at the above-mentioned stereotaxic levels was estimated, as was the number of cells in each area (cells/mm^3^). GFAP, Iba-1, and Hoechst-stained area was calculated as the mean value obtained from the four different images. Hoechst-stained nuclei were used to calculate the percentage of GFAP- or Iba-1-positive cells in each image (% of astrocytes or microglia of the total nuclei), and the Hoechst-stained area was used to calculate the percentage of GFAP- and Iba-1-positive area in each image.

### Statistical Analysis

Data are expressed as mean ± SEM. The equilibrium dissociation constant of unlabeled ligands was calculated by measuring their competition for radioligand binding. Microglial and astrocyte immunoreactivities as well as the percentages of [^3^H]CP55,940 and [^35^S]GTPγS binding stimulation evoked by the agonists were analyzed by a two-tailed unpaired Student’s *t*-test. The statistical analyses were performed using GraphPad Prism 5.01 software. The threshold for statistical significance was set at *p* < 0.05.

## Supporting Information Available

Percentage of inhibition of [^3^H]CP55,940 binding in the presence of CB_1_ and CB_2_ antagonists in a rat lesion model of AD; [^3^H]CP55,940 binding in a rat lesion model of AD; cannabinoid receptor coupling to G_i/0_-proteins evoked by CP55,940 and WIN55,212-2 in control rats; Iba-1 and iNOS immunostaining in aCSF and SAP groups; acetylcholinesterase enzymatic staining assay performed in aCSF and SAP groups, as a validation of the BFCN lesion; and raw data from the autoradiographic studies (PDF)

## Abbreviations

ACEAarachidonyl-2-chloroethylamideAD: arachidonyl-2-chloroethylamideADAlzheimer’s disease
B: nucleus basalis magnocellularis
BFCNs: basal forebrain cholinergic neurons
BSA: bovine serum albumin
CNS: central nervous system
Cx: cortex
DTT: dl-dithiothreitol
eCB: endocannabinoid system
GTPγS: guanosine 5′-O-3-thiotriphosphate
HPC CA3: hippocampus CA3 area
HPC DG: hippocampus dentate gyrus
MS: multiple sclerosis
MWM: Morris water maze
NORT: novel object recognition test
N: nuclei
PA: passive avoidance
ROI: region of interest
ROS: reactive oxygen species
TBI: traumatic brain injury
WT: wild-type
