# Supplementary material for: Cannabinoid Receptors and Glial Response Following a Basal Forebrain Cholinergic Lesion
Source: ACS Pharmacol Transl Sci. 2022 Aug 4;5(9):791–802. doi: 10.1021/acsptsci.2c00069 (PMC9469185; doi:10.1021/acsptsci.2c00069)
Supplement: Supplementary file 1 — pt2c00069_si_001.pdf [file pt2c00069_si_001.pdf]

Supporting Information

Cannabinoid receptors and glial response following a basal forebrain cholinergic lesion

Alberto Llorente-Ovejero[1#], Iker Bengoetxea de Tena[1#], Jonatan Martínez-Gardeazabal[1,2], Marta Moreno-Rodríguez[1], Laura Lombardero[1], Iván Manuel[1,2], Rafael Rodríguez-Puertas[1,2*]

[1]Department of Pharmacology, University of the Basque Country (UPV/EHU), Leioa, Spain

[2]Neurodegenerative diseases, Biocruces Bizkaia Health Research Institute, Barakaldo, Spain

#Alberto Llorente-Ovejero and Iker Bengoetxea de Tena contributed equally to this article.

[*]**Correspondence:**

Rafael Rodríguez-Puertas

rafael.rodriguez@ehu.es

Supporting Information

Percentage of inhibition of [^{3}H]CP55,940 binding in presence of CB_1 or CB_2 antagonists in a rat lesion model of AD

In all four analyzed areas, the SR141716A specific CB_1 receptor antagonist almost completely blocked binding in a dose-dependent manner, with inhibition reaching approximately 90-100%, depending on the area, with a concentration of 1 μM, in both aCSF and SAP groups (see figure). To the contrary, specific CB_2 receptor antagonist SR144528 was able to inhibit binding up to 25%, at best (see figure).

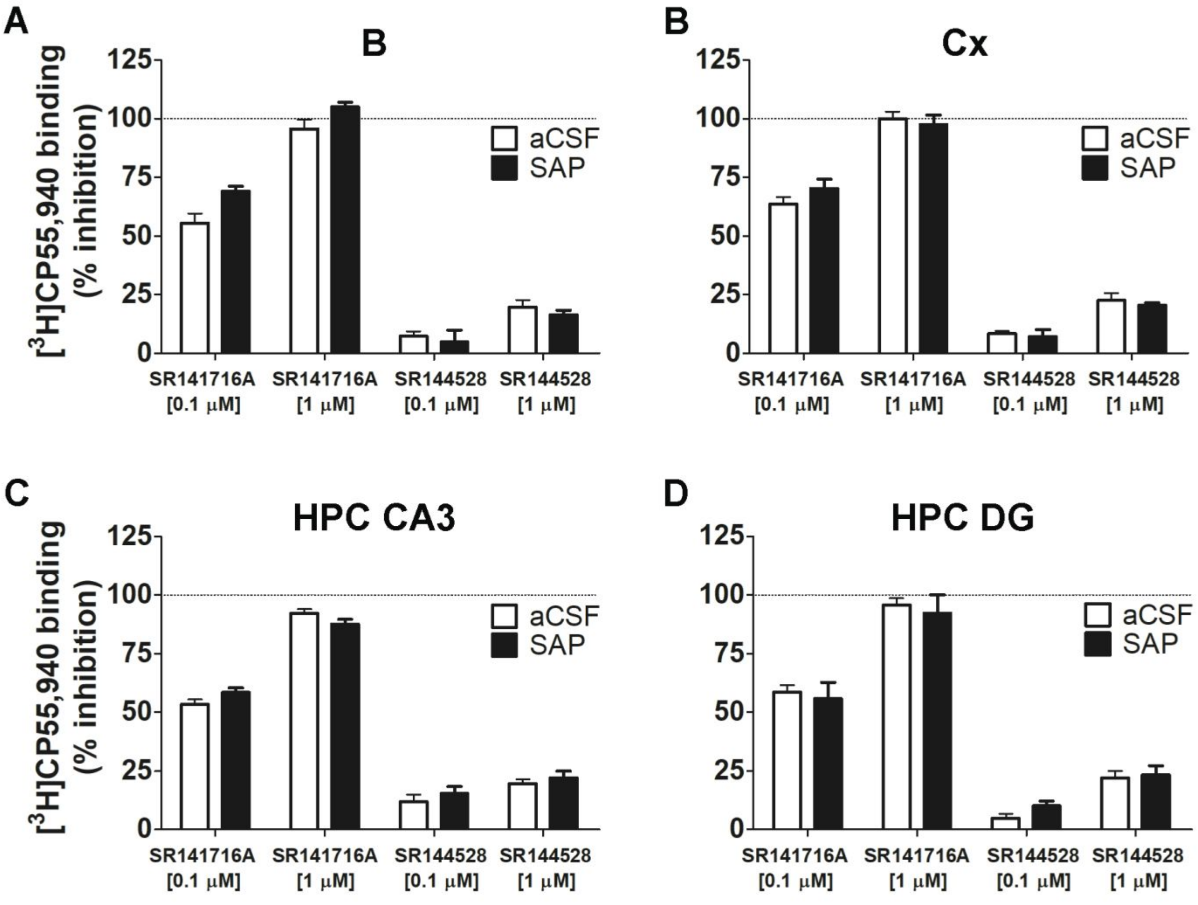

Figure S1. Percentage of inhibition of [^{3}H]CP55,940 binding in the presence of specific antagonists, SR141716A and SR144528, in different brain regions of aCSF (n = 8) and SAP (n = 8) Sprague-Dawley rats: **A)** B, **B)** Cx, **C)** HPC CA3 and **D)** HPC DG. No differences were observed between aCSF and SAP groups. Cortex: Cx; *nucleus basalis magnocellularis*: B; hippocampus dentate gyrus: HPC DG; Hippocampus CA3 area: HPC CA3.

CB_1 receptor density ([^{3}H]CP55,940 binding) in aCSF and SAP groups

CB_1 receptor density increased in the B and decreased in the HPC, in CA3 and DG regions, following the 192IgG-saporin-induced lesion of BFCN. No changes were observed in the cortex.

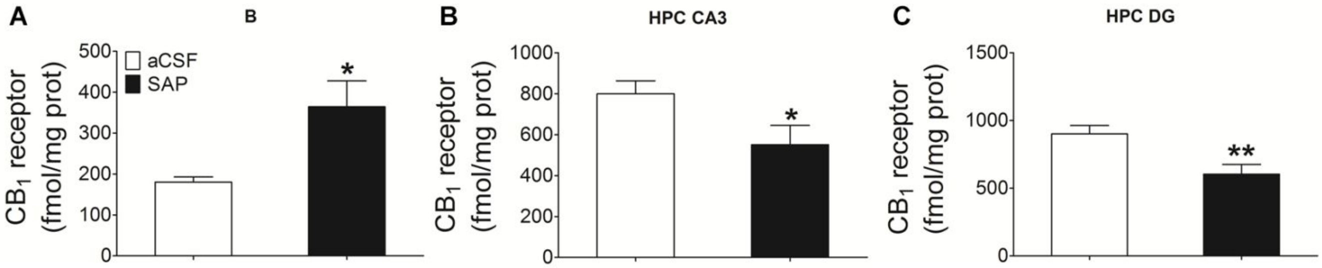

Figure S2. [^{3}H]CP55,940 binding in different brain regions of aCSF (n = 8) and SAP (n = 8) Sprague-Dawley rats: **A)** B, **B)** HPC CA3 and **C)** HPC DG. No changes were observed in the cortex. $^*p<0.05$ and $^{**}p<0.01$, aCSF vs. SAP. *Nucleus basalis magnocellularis*: B; hippocampus dentate gyrus: HPC DG; Hippocampus CA3 area: HPC CA3.

Cannabinoid receptor coupling to $G_{i/0}$-proteins evoked by CP55,940 and WIN55,212-2

In all four analyzed areas, no significant differences were found between cannabinoid receptor coupling to $G_{i/0}$-proteins evoked by CP55,940 and WIN55,212-2. However, although not statistically significant, WIN55,212-2 evoked slightly higher coupling to $G_{i/0}$-proteins in all four analyzed areas.

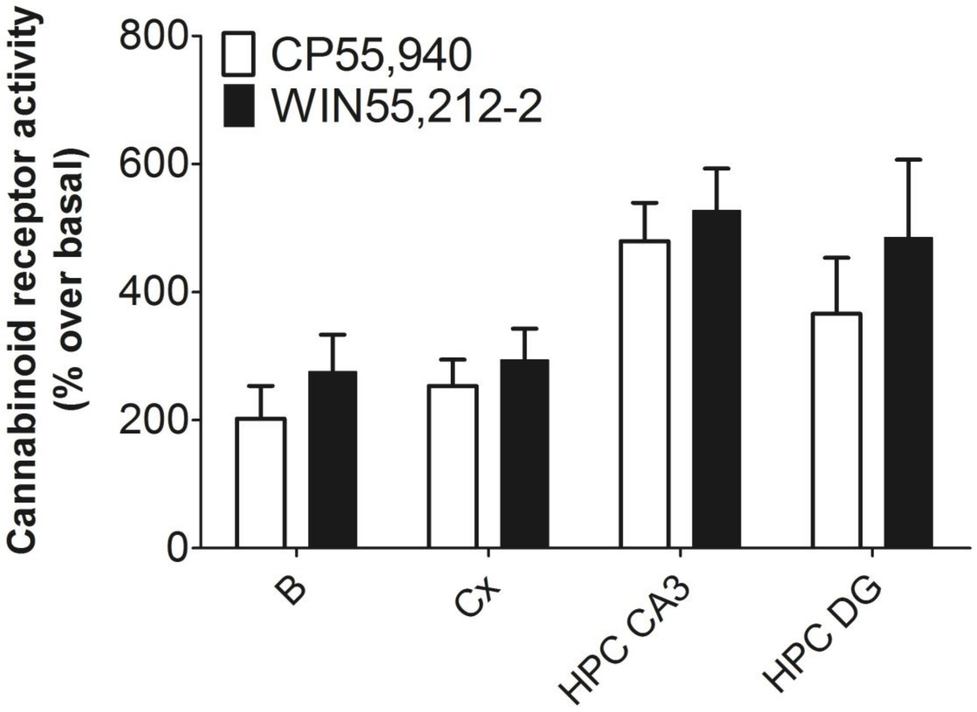

Figure S3. Cannabinoid receptor coupling to $G_{i/0}$-proteins ([^{35}S]GTPγS binding) evoked by CP55,940 and WIN55,212-2 in different brain regions of control Sprague-Dawley rats: B, Cx, HPC CA3 and HPC DG. No statistically significant differences were observed between the cannabinoid receptor activities evoked by both agonists. Cortex: Cx; *nucleus basalis magnocellularis*: B; hippocampus dentate gyrus: HPC DG; Hippocampus CA3 area: HPC CA3.

Iba-1 and iNOS immunostaining in aCSF and SAP groups

Note the increased colocalization of Iba-1 and iNOS immunostaining at the lesion site (B) in SAP group compared to control aCSF group. Similarly, comparing different areas in SAP group, note the increased colocalization at the lesion site (B) compared to cortical projection areas (Cx). This may be indicative of differential microglial phenotypes (higher proportion of M1 microglia) at the lesion site. However, a precise detection and quantification of microglial phenotypes requires specific studies, including transcriptomic approaches, which fall beyond the scope of the present work and shall be conducted in following studies.

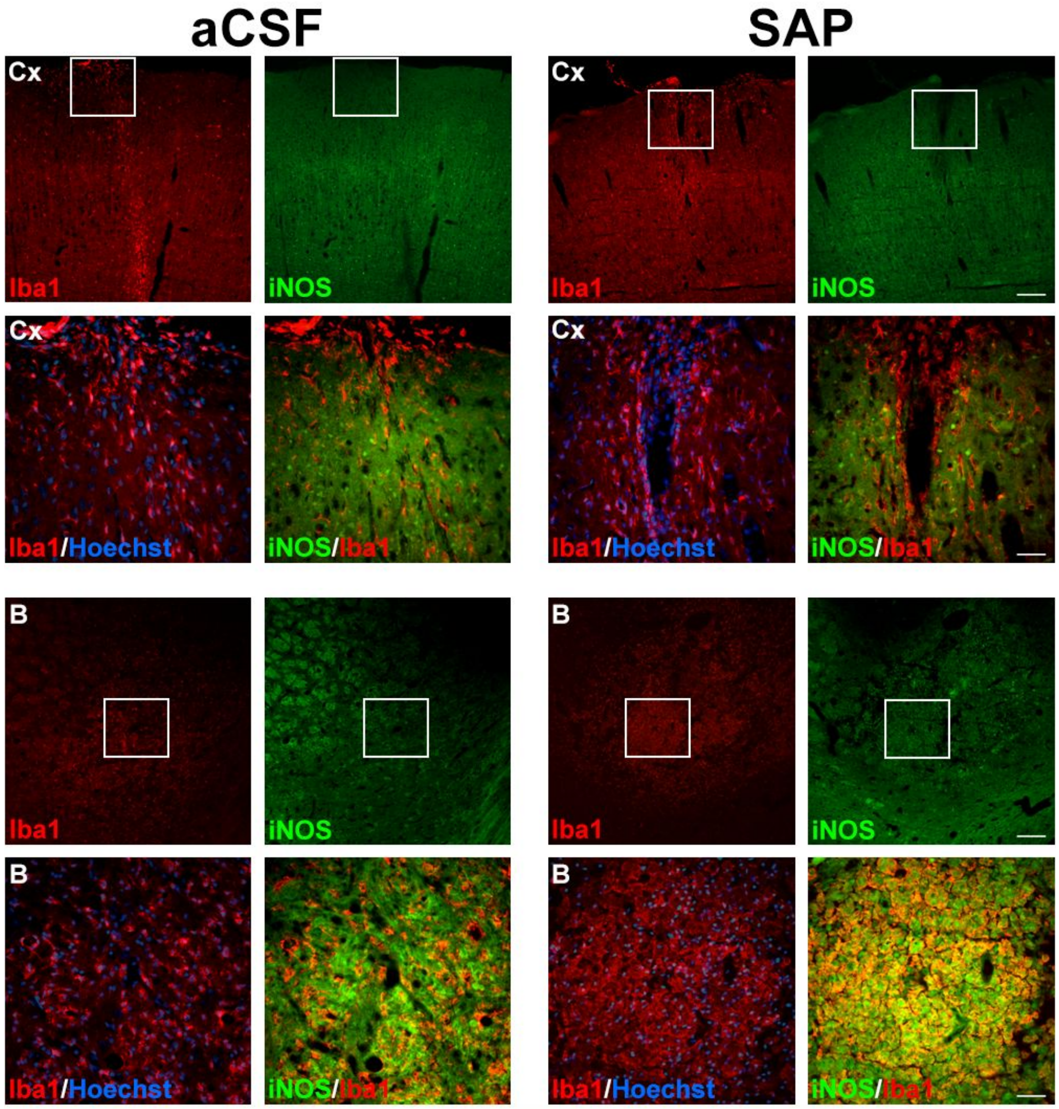

Figure S4. Top photographs from each block correspond to 100-fold magnification images (scale bar = 200 μm) of Iba-1 (left) and iNOS (right) stained slices of aCSF and SAP Sprague-Dawley rats. Squares indicate the areas used shown in the magnified images. Bottom photographs from each block correspond to 400-fold magnification (scale bar = 50 μm) merged images of Hoechst and Iba-1 staining (left) and iNOS and Iba-1 staining (right) stained slices of aCSF and SAP Sprague-Dawley rats. *Cortex: Cx; nucleus basalis magnocellularis: B.*

Acetylcholinesterase enzymatic staining in aCSF and SAP groups

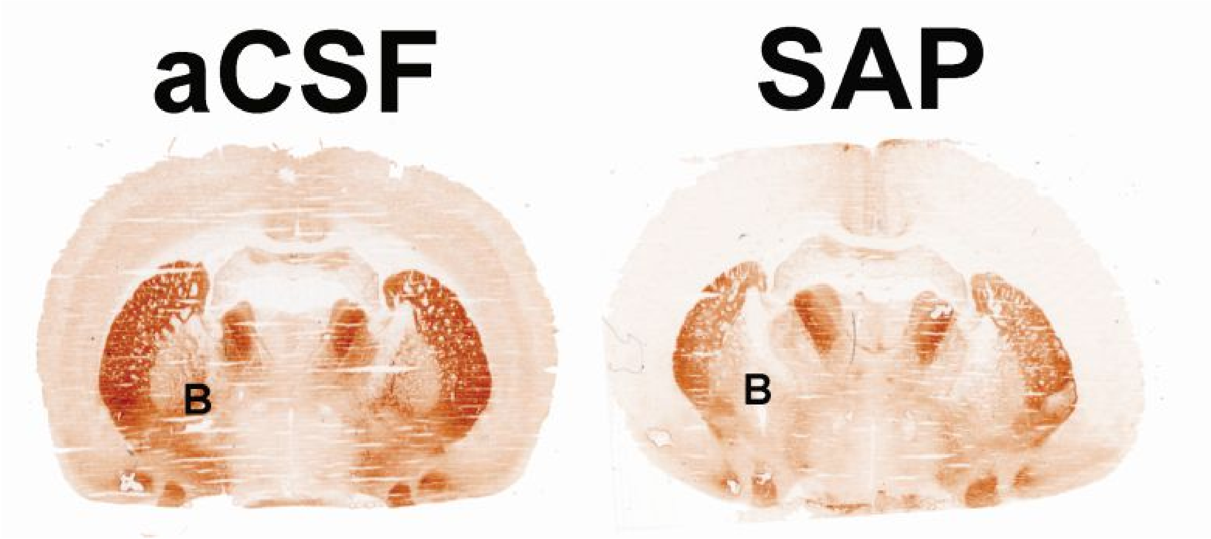

Figure S5. Representative images from acetylcholinesterase enzymatic staining in the B of aCSF and SAP Sprague-Dawyley rats. Note the decrease in acetylcholinesterase staining in the B, as well as in cortical projection areas, following the lesion (SAP group).

Raw data from the autoradiographic studies shown in Figures 5 and 6

Data from Figure 5

				CP55,940 (10 μM)		HU308 (10 μM)	
Area	Condition	Animal	Basal	nCi/mg tissue	% over basal	nCi/mg tissue	% over basal
Cx	aCSF	R1	756,51	1094,00	44,61	1010,28	33,54
		R2	634,93	980,00	54,35	879,00	38,44
		R3	1044,42	1295,58	24,05	1235,00	18,25
		R4	856,17	1360,12	58,86	1159,03	35,37
		R5	888,98	1404,00	57,93	1197,00	34,65
	SAP	R13	764,96	1282,66	67,68	1322,00	72,82
		R14	753,31	1275,00	69,25	1159,00	53,85
		R16	770,13	1313,42	70,55	1567,56	103,55
		R19	611,33	1227,54	100,80	1330,20	117,59
		R21	875,47	1285,00	46,78	1438,50	64,31
HPC CA1	aCSF	R1	723,47	1206,08	66,71	1251,53	72,99
		R2	666,00	1224,91	83,92	1511,44	126,94
		R3	744,90	882,40	18,46	1272,39	70,81
		R4	855,10	1176,08	37,54	1087,73	27,20
		R5	826,21	1341,05	62,31	1514,35	83,29
	SAP	R13	636,21	973,07	52,95	1027,49	61,50
		R14	738,40	1283,75	73,86	1437,99	94,75
		R16	706,35	1225,13	73,44	1199,86	69,87
		R19	738,91	1076,41	45,67	1374,16	85,97
		R21	622,42	1116,95	79,45	1369,07	119,96
HPC CA3	aCSF	R1	732,47	1306,27	78,34	1408,93	92,35
		R2	655,00	1369,81	109,13	1181,22	80,34
		R3	719,90	1020,07	41,70	1044,95	45,15
		R4	706,10	1025,00	45,16	1196,51	69,45
		R5	773,70	1294,99	67,38	1615,84	108,85
	SAP	R13	666,05	1041,74	56,41	1013,18	52,12
		R14	635,74	1158,96	82,30	1090,80	71,58
		R16	664,74	1343,54	102,12	1126,21	69,42
		R19	783,25	1141,64	45,76	1353,74	72,84
		R21	648,50	1049,34	61,81	1083,32	67,05

				CP55,940 (10 µM)		HU308 (10 µM)	
Area	Condition	Animal	Basal	nCi/mg tissue	% over basal	nCi/mg tissue	% over basal
HPC DG	aCSF	R1	551,47	1138,46	106,44	959,40	73,97
		R2	484,00	1074,04	121,91	996,47	105,88
		R3	542,90	1058,19	94,91	934,08	72,05
		R4	540,10	850,38	57,45	821,77	52,15
		R5	555,83	1161,00	108,88	1102,19	98,29
	SAP	R13	518,70	883,42	70,31	833,56	60,70
		R14	586,00	913,79	55,94	761,51	29,95
		R16	573,30	1074,71	87,46	877,49	53,06
		R19	626,87	816,79	30,30	967,54	54,34
		R21	504,86	927,43	83,70	795,21	57,51
Glob pallidus	aCSF	R1	1533,11	10573,09	589,65	3778,63	146,47
		R2	1833,44	9076,04	395,03	3834,46	109,14
		R3	1416,58	8844,52	524,36	2479,59	75,04
		R4	1921,39	9805,20	410,32	2502,14	30,23
		R5	2181,28	9963,25	356,76	5492,18	151,79
	SAP	R13	1015,33	7719,00	660,25	2242,33	120,85
		R14	1686,25	6968,36	313,25	3557,88	110,99
		R16	1658,90	7448,68	349,01	3215,60	93,84
		R19	1821,16	7496,42	311,63	3872,52	112,64
		R21	1081,19	6327,87	485,27	2937,10	171,66
B	aCSF	R1	1011,57	1809,00	78,83	1939,52	91,73
		R2	1285,00	2187,00	70,19	2166,46	68,60
		R3	833,87	2443,15	192,99	2037,61	144,36
		R4	851,79	1614,31	89,52	1568,00	84,08
		R5	988,71	1660,60	67,96	1739,65	75,95
	SAP	R13	1075,00	2093,00	94,70	1593,00	48,19
		R14	890,00	1720,36	93,30	1927,00	116,52
		R16	874,00	1610,00	84,21	1404,36	60,68
		R19	1058,00	1474,00	39,32	1399,95	32,32
		R21	1157,00	2060,00	78,05	2092,00	80,81
Striatum	aCSF	R1	918,29	1459,72	58,96	1466,06	59,65
		R2	765,10	1899,59	148,28	1325,79	73,28
		R3	751,80	1351,00	79,70	1303,99	73,45
		R4	1112,63	1720,20	54,61	1355,40	21,82
		R5	1123,33	2216,89	97,35	1480,18	31,77
	SAP	R13	905,74	1662,31	83,53	1205,10	33,05
		R14	1019,19	1392,70	36,65	1534,32	50,54
		R16	918,90	2094,68	127,96	1701,00	85,11
		R19	1165,33	1934,00	65,96	1614,84	38,57
		R21	964,80	1365,00	41,48	1470,11	52,37

Figure S6. Raw data corresponding to the [^{35}S]GTPγS binding from figure 5. Basal [^{35}S]GTPγS binding (nCi/g t.e.), CP55,940 (10 µM) evoked stimulation (percentages) and HU308 (10 µM) evoked stimulation. *Cortex: Cx; nucleus basalis magnocellularis: B; hippocampus dentate gyrus: HPC DG; hippocampus CA3 area: HPC CA3; hippocampus CA1 area: HPC CA1; Glob pallidus; Globus pallidus.*

Data from Figure 6

Area	Condition	Animal	Basal	CP55,940 (10 µM)		HU308 (10 µM)		CP55,940 (10 µM) + SR141716A (1 µM)	
				nCi/mg tissue	% over basal	nCi/mg tissue	% over basal	nCi/mg tissue	% over basal
Cx	SAP	R13	764,96	1282,66	67,68	1322,00	72,82	527,04	-31,10
		R14	753,31	1275,00	69,25	1159,00	53,85	532,76	-29,28
		R16	770,13	1313,42	70,55	1567,56	103,55	553,10	-28,18
		R19	611,33	1227,54	100,80	1330,20	117,59	534,37	-12,59
		R21	875,47	1285,00	46,78	1438,50	64,31	630,10	-28,03
HPC DG	SAP	R13	518,70	883,42	70,31	833,56	60,70	485,33	-6,43
		R14	586,00	913,79	55,94	761,51	29,95	444,97	-24,07
		R16	573,30	1074,71	87,46	877,49	53,06	615,14	7,30
		R19	626,87	816,79	30,30	967,54	54,34	568,12	-9,37
		R21	504,86	927,43	83,70	795,21	57,51	481,33	-4,66
B	SAP	R13	1075,00	2093,00	94,70	1593,00	48,19	709,11	-34,04
		R14	890,00	1720,36	93,30	1927,00	116,52	662,86	-25,52
		R16	874,00	1610,00	84,21	1404,36	60,68	601,65	-31,16
		R19	1058,00	1474,00	39,32	1399,95	32,32	521,17	-50,74
		R21	1157,00	2060,00	78,05	2092,00	80,81	561,00	-51,51

Area	Condition	Animal	Basal	CP55,940 (10 µM) + SR144528 (1 µM)		HU308 (10 µM) + SR141716A (1 µM)		HU308 (10 µM) + SR144528 (1 µM)	
				nCi/mg tissue	% over basal	nCi/mg tissue	% over basal	nCi/mg tissue	% over basal
Cx	SAP	R13	764,96	1334,00	74,39	606,84	-20,67	1010,85	32,14
		R14	753,31	1264,00	67,79	647,91	-13,99	1017,43	35,06
		R16	770,13	1131,44	46,92	638,26	-17,12	1088,25	41,31
		R19	611,33	1193,27	95,19	658,07	7,65	737,06	20,57
		R21	875,47	1254,02	43,24	727,22	-16,93	1144,01	30,67
HPC DG	SAP	R13	518,70	1001,71	93,12	490,00	-5,53	703,40	35,61
		R14	586,00	1058,66	80,66	376,46	-35,76	834,61	42,43
		R16	573,30	995,79	73,69	530,43	-7,48	831,26	45,00
		R19	626,87	966,93	54,25	565,00	-9,87	671,77	7,16
		R21	504,86	861,60	70,66	633,37	25,45	636,06	25,99
B	SAP	R13	1075,00	1401,36	30,36	887,99	-17,40	1505,64	40,06
		R14	890,00	1296,26	45,65	751,40	-15,57	1324,01	48,77
		R16	874,00	1966,00	124,94	691,92	-20,83	1034,58	18,37
		R19	1058,00	1436,00	35,73	671,00	-36,58	986,00	-6,81
		R21	1157,00	1612,00	39,33	739,00	-36,13	1625,00	40,45

Figure S7. Raw data corresponding to the [^{35}S]GTPγS binding from figure 6. Basal [^{35}S]GTPγS binding (nCi/g t.e.), CP55,940 (10 µM) evoked stimulation (percentages) and HU308 (10 µM) evoked stimulation, as well as stimulation evoked in the presence of specific CB_1 and CB_2 antagonists, SR141716A and SR144528. *Cortex: Cx; hippocampus dentate gyrus: HPC DG; nucleus basalis magnocellularis: B.*
